# Metabolic and behavior changings during the transition period as predictors of calving proximity and welfare of dairy goats

**DOI:** 10.1016/j.vas.2021.100168

**Published:** 2021-01-22

**Authors:** Manuela Silva Libânio Tosto, Stefanie Alvarenga Santos, Roberto da Costa Pinto Filho, Thomaz Cyro Guimarães de Carvalho Rodrigues, Isis Miranda Carvalho Nicory, Gleidson Giordano Pinto de Carvalho, Rodrigo Freitas Bittencourt, Maria Consuelo Caribé Ayres, Taiala Cristina de Jesus Pereira

**Affiliations:** School of Veterinary Medicine and Animal Science, Federal University of Bahia, Salvador, Bahia, Brazil

**Keywords:** Animal welfare, negative energy balance, Social behavior, Transition period

## Abstract

This Research aimed to evaluate the metabolic status and behavior changes during the transition period in dairy goats from three breeds, under tropical conditions. Thirty multiparous female goats were kept in pens, distributed randomly by breeds. Infrared cameras were fitted in the pens to monitor the animals, and its activities were recorded. Goats displayed varied idle, standing, walking, and feeding behaviors at kidding day (*P* < 0.10) when compared with the days after and before. Agnostic interactions prevailed between 3.33 and 7.98% of the time on the day of kidding. There was a day effect for the exploratory activities (*P* < 0.10), where the most exploratory activities were observed on the day of kidding. The milk production and fat content differed according to breed and lactation week. Collective pens for lactating goats, kept in a tropical environment, do not compromise the social behavior and welfare.

## 1 Introduction

The transition period is considered critical for dairy goats (Matthews, 2016; Stelletta, Gianesella & Morgante, 2008). When the kidding period approaches, the risk of metabolic diseases is particularly high. There is substantial increase in energy requirements to support pregnancy and lactation when feed intake decrease around this period (Sundrum, 2015; Zamuner, DiGiacomo, Cameron & Leury, 2020). In addition, the production level of the genetic group can also affect these requirements (Radin, Simpraga, Vince, Kostelić & Milinković-Tur, 2015).

Negative energy balance (NEB) can be established when energy demand for physiological functions exceeds the nutrients intake, which could cause mobilization of triglycerides from adipose tissue for cellular oxidation. This state may be worsening in the genetic groups that are better adapted to milk production since they respond more effectively to homeothetic alterations for lactogenesis (Bauman & Currie, 1980). During the transition period, a severe NEB may lead to a direct or indirect disturbance due to the altered metabolism of energy, protein, and minerals.

The metabolic profile of blood is a common tool for monitoring possible metabolic changes. This profile helps evaluate the subclinical disorders that may affect lactating females (Chapinal et al., 2011; Leblanc et al., 2006) and can help design preventive protocols for dairy goats. However, alternative and non-invasive methods, such as feed and social behavior, as well as movement patterns, can be used to monitor metabolic disorders during the transition period, and to predict if kidding is near (Frost et al., 1997; González, Tolkamp, Coffey, Ferret & Kyriazakis, 2008).

The study of animal behavior is considered one of the main welfare markers, and it can be used to identify the animals that are adapted to different climates (Metz & Wierenga, 1997). Environmental factors, such as climate, social interactions, and pen density, can influence the goat behavior. In addition, the physiological phase and homeorrhetic state can alter behavior patterns and negatively affect animal welfare.

Therefore, correct understanding and characterization of both changes, during, and after kidding, are important to accurate predict and prevent the NEB-related metabolic disorders (Matthews, 2016; Overton, McArt & Nydam, 2017). Thus, the aims of this study were (1) to evaluate temporal variations in circulating levels of selected metabolites involved in the energetic metabolism regulation during the transition period and (2) to investigate the social behavior changes in periparturient dairy goats from three genetic groups kept under tropical conditions.

## 2 Materials and methods

### 2.1 Ethical considerations

All the animal care and handling procedures were approved by the Ethics Committee on Animal Use of the School of Veterinary Medicine and Animal Science (Federal University of Bahia – UFBA), with protocol number 27/2014.

### 2.2 Local, animals, experimental design and diets

The experiment was conducted at the Experimental Farm of the School of Veterinary Medicine and Animal Science – UFBA, located in the city of Entre Rios, State of Bahia, Brazil. Thirty multiparous female goats were used, as follows: 10 breed Saanen, 10 Anglo-Nubian, and 10 Moxotó (average two-years-old). The animals were kept at six collective pens, with five goats distributed for each one. They were separated from breed, being two pens for each breed. In the end, we had two pens for Saanen, two for Anglo-Nubian, and two for Moxotó goats.

The pen's size was 10.5 m^2^. All pens were equipped with collective feeders of 3.5 linear meters, automatic 7-liter drinkers, and infrared monitoring cameras (model cd-1030, JFL alarms). The occupational density was 2.1 m^2^ per animal in each pen. All the animals were submitted to a 10-days adaptation period, during which they were weighed, identified, dewormed, and vaccinated. After adaptation, all the animals were submitted to an estrus synchronization protocol.

After the reproductive protocol, 24 goats presented a positive gestation diagnosis, being considered then the 24 experimental units. However, all 30 animals were kept in pens to maintain the initial occupational density (5 goats per pen). Of the total number of pregnant goats, 9 were of the Saanen breed (initial body weight, IBW: 35.9 ± 1.3 kg), 8 of the Moxotó breed (IBW: 25.9 ± 1.9 kg), and 7 of the Anglo-Nubian breed (IBW: 42.6 ± 1.6 kg). For the metabolic evaluation the animals were separated by breed, then randomly distributed, with three breeds taken as the experimental treatments to evaluate metabolic changes, with 9, 8, and 7 replications for Saanen, Moxotó, and Anglo-Nubian, respectively.

From the 100-day gestation period to kidding, animals were fed with diet 1(Table 1) to meet the requirements of mature gestation with twin kids following the recommendations of the National Research Council (NRC, 2007), using 70:30 as the forage to concentrate ratio (Table 1). This diet contained 110 g of crude protein (CP) per kg of dry matter (DM). After kidding, diet 2 was formulated to meet the requirements of lactating goats with a 1.5-liter of daily milk production, according to the recommendations of the NRC (2007). The forage to concentrate ratio was 60:40 (Table 1), and the CP content was 135 g of CP per kg of DM.

**Table 1:** Chemical composition of corn and sorghum silage, concentrates and diets.

Chemical composition, g/kg DM	Silage	Concentrate	Diet 1	Diet 2
			(70S:30C)	(60S:40C)
Dry matter^1^	318	943	505	566
Organic matter	947	929	941	947
Crude Protein	56.9	249	98.7	94.4
Ether extract	26.0	23.9	25.9	27.7
NDFap^2^	522	838	438	397
ADFap^3^	274	822	213	187
Non-fiber carbohydrates	342	573	379	430
Lignin	53.0	19.9	42.1	37.3

S silage; C concentrate; ^1^ g/kg of natural matter, ^2^Neutral detergent fiber corrected for ashes and protein; ^3^Acid detergent fiber corrected for ashes and protein.

The animals were fed twice daily at 0730 h and 1500 h in similar proportions, after milking. Milking occurred once daily using a portable milking machine (Model Eurovac 3500 EuroLatte) at 0530 h. Additional concentrate supplementation (220 g CP/kg DM – Table 1) was made for goats that produced more than 900 g of milk, with 4% of fat. The addition of 100 g of concentrate (the same of the diets) was included in the goat diet for each 300 g of milk produced during the morning milking session. This was based on the milk production of the previous week. Intake was adjusted to maintain a maximum of 15% leftovers of fresh feed.

### 2.3 Monitoring animal behavior

Images were taken continuously for each behavior category to establishing the activities. The images of three consecutive days per animal were used: prepartum (one day before kidding), kidding (on the day of kidding) and postpartum (one day after kidding), with the following daytime intervals: T1 = 0500–0630 h, T2 = 0800–1030 h, T3 = 1200–1330 h, T4 = 1530–1700 h and T5 = 1700–2030 h, which totalled approximately 1030 h of image recordings per animal/day.

Different types of behavior were described in relation to video image identification and frequency of each observation. The following activities of the animals were identified and recorded: resting, walking and standing time, agnostic and non-agnostic interaction, exploratory, self-grooming, rumination, and feeding time.

### 2.4 Collectingg blood samples and milk production

Immediately at kidding (day 0) and for more 56 days after, with 7-day intervals, blood samples were collected. This totalled 9 blood samples collections per animal at the end of the 56 days’ period. Samples were collected before morning feeding via jugular venepuncture in tubes without anticoagulant. After the venepuncture, the samples were immediately centrifuged at 750 × *g* for 15 min to extract blood serum, and aliquots were preserved into Eppendorf and stored at - 20 °C for subsequent laboratory analyses.

Milk production was measured every 15 days, with the first measurement taken 15 days after kidding, and finished after 45 days, totalling 3 measurements. Milking was performed manually and milk production was weighed on a digital scale (model AS-110, Elgin). Milk samples of each goat were taken after homogenization, preserved with bronopol, and sent to the Milk Laboratory of ESALQ-USP/ Piracicaba, São Paulo for later analyses. Samples were analysed by the IR methodology (Lactoscan®; Entelbra, SP, Brazil).

### 2.5 Environmental characterization and physiological parameters

**Fig. 1:**
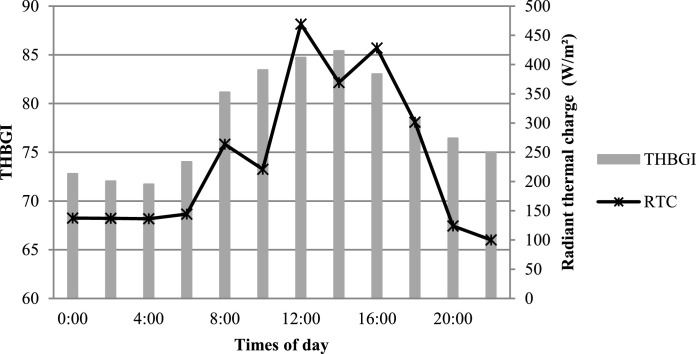
Vermon globe temperature and humidity index (THBGI) measurements and radiant thermal capacity (W/m^2^).

The environmental variables in pens were monitored on the day of kidding and at 14, 28, 42, and 56 days postpartum. On each day, the measurements of the environmental variables were taken at specific times: 0000 h; 0200 h; 0400 h; 0600 h; 0800 h; 1000 h; 1200 h; 1400 h; 1600 h; 1800 h; 2000 h; 2200 h. The following variables were measured: ambient temperature (AT), black globe temperature (BGT), wind speed (WS), and relative humidity (RH) using a Thermo hygrometer, an anemometer, and black globe temperature sensor. The temperature and humidity index of the black globe (THBGI) was determined according to the equation described by Buffington et al. (1977): THBGI = Tdb + 0.36Tdt - 330.08, where Tdb = dry bulb temperature and Tdt = dew point temperature. The radiant thermal charge (RTC) was calculated by the equation: RTC = 1053 hc (Tbg - Ta) + σ Tbg^4^, W/m^2^, where: hc = black globe convection coefficient, W/m^2^/k; Tbg = temperature of the globe thermometer, ^°^ K; Ta = ambient temperature, ^°^ K; σ = the Stephan-Boltzman constant (5, 6697 × 10^−8^, W/m²/k^4^). The obtained means for THBGI and RTC are shown in Fig. 1.

### 2.6 Laboratory analysis

Chemical analyses of the ingredients and experimental diets were carried out according to the analytical procedures of the Official methods of analysis of AOAC International (AOAC, 2005) following the grinding of the samples in Wiley knife mill (A.H. Thomas, Philadelphia, PA) with a 1-mm sieve. The dry matter (DM, method 934.01), crude protein (CP, method 981.10), ether extract (EE, method 920.29), and ash (method 942.05) contents were determined.

The neutral detergent fiber (NDFap) was measured by treating samples with thermostable alpha-amylase, without the use of sodium sulphite. Results were corrected for residual ash according to the methodology proposed by Mertens et al. (2002) and N residues (Licitra, Hernandez & Van Soest, 1996). For the determination of acid detergent lignin (ADL), the acid detergent fiber (ADF) residue was treated with 72% sulphuric acid based on the methodology described by Detmann et al. (2012), in which ADF residue was obtained by sequential analysis. The quantification of non-fibrous carbohydrates (NFC) was obtained from the model of Hall (2000).

Glucose (GL), total cholesterol (TC), albumin, total protein, calcium, phosphorus, and urea were quantified with spectrophotometer readings (Multiskan GO Microplate Spectrophotometer, Thermo scientific). Glucose concentrations were quantified by the enzymatic method. The total cholesterol concentrations were quantified by the enzymatic colorimetric methodology. The albumin concentrations were quantified by the green bromocresol methodology. The total protein (PrT) concentrations were quantified by the biuret methodology. The total calcium concentration was quantified by the colorimetric method (cresolphthalein - CPC). The magnesium concentration, inorganic phosphorus, and urea were quantified by the colorimetric method. All laboratory analyses were made with a commercial *kit* (Labtest, MG, Brazil).

The serum concentrations of aspartate-aminotransferase (AST), gamma-glutamyltransferase (GGT), triglycerides (TRI), and high-density lipoprotein (HDL) cholesterol were measured by the semiautomatic biochemical analyzer (Bioplus 2000, SP, Brazil). The AST activity was determined by the UV kinetic; the GGT was determined by the colorimetric kinetic; the triglyceride concentrations were quantified by the enzymatic system; the direct HDL cholesterol concentrations were determined by the direct colorimetric enzymatic. All these analyses were determined using a commercial kit (Doles, GO, Brazil).

The beta-hydroxybutyrate (BHBA) analyses were carried out in the Biochemistry and Animal Physiology laboratory at the University of São Paulo, SP, Brazil. The BHBA concentrations were determined by the colorimetric endpoint and kinetic enzyme method using a commercial kit (RANDOX®, Crumlin, England). Readings were taken using an automatic blood biochemistry analyzer (SBA-200, CELM, SP, Brazil).

### 2.7 Statistical analyses

The behavior data were analysed by the GLIMMIX procedure of SAS (version 9.2), with the fixed effects related to kidding (one day before, kidding day and one day after), schedule (daytimes intervals - T1, T2, T3, T4, and T5), and the interaction between them. The pen was considered to be a random effect in the model. Schedules were considered time-repeated measures and the following continuous probability distributions were tested for each variable: exponential, log-normal, gamma, Weibull, t-distribution, inverse Gaussian and normal. The criteria for obtaining the best fit for these distributions were the criterion of maximum likelihood, and the relationship between Chi-square and degrees of freedom, which were better the greater the proximity of 1. The Tukey test was used to compare means. All the analyses were conducted by taking 0.10 as the critical level of probability for type I error, through the following statistical model:

$$Y_{ijkl}=\;\mu +K_{i}+\delta_{ij}+{\left(K^{*}T\right)}_{ik}+p_{l}+\;\varepsilon_{ijkl}$$

Where: Y_ijkl_ = observation ijkl, μ = overall mean, *K* = fixed effect of kidding (*i* = 3), δ = random error kidding effect; *T* = fixed effect of schedule (*k* = 5), K*T = interaction effect between fixed effects kidding and schedule, *p* = random effect of pen (*l* = 6) and ε = random error within each subject.

The blood metabolites and physiological parameters were analysed by the GLIMMIX procedure of SAS (version 9.2). Breed, lactation (days in milking), and the interaction between them were considered fixed effects in the statistical model. The pen was considered a random effect. The other procedures were identical to those performed above, and Tukey's test was used to compare means. The following model was used:

$$Y_{ijkl}=\;\mu +G_{i}+\delta_{ij}+{\left(G^{*}L\right)}_{ik}+p_{l}+\;\varepsilon_{ijkl}$$

Where: Y_ijkl_ = observation ijkl, μ = overall mean, *G* = fixed effect of breed (*i* 3), δ = random error associated to breed; *L* = fixed effect of lactation (days in milking) (*k* = 9), G*L = interaction effect between fixed effects breed and lactation (days in milking), *p* = random effect of pen (*l* = 6) and ε = random error within each subject.

## 3 Results

**Fig. 2:**
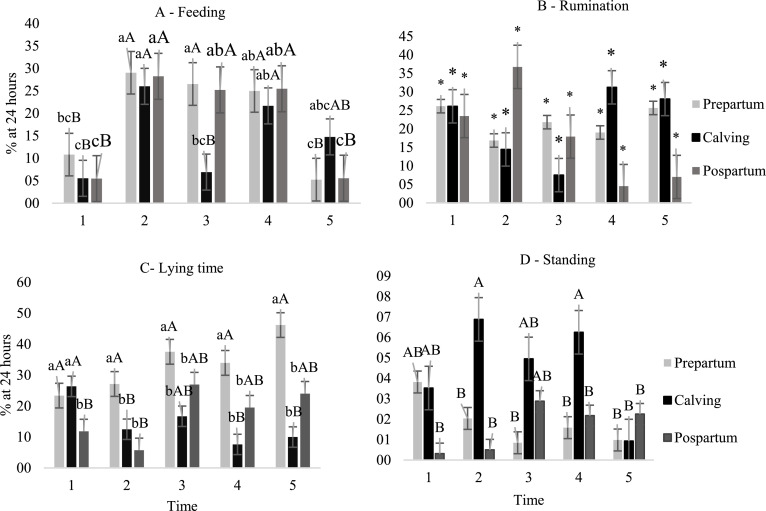
Behavior of goats evaluated one day before kidding on the day of kidding and one day after kidding at different times of the day: 1 (05:00–6:30 h); 2 (08:30–10:00 h); 3 (12:00–13:30 h); 4 (15:30–17:00 h) and 5 (19:00–20:30 h). Feeding (A); rumination (B); lying time (C); standing (D), time expressed as a percentage of 24 h. Lowercase letters differ for times and uppercase letters differ for kidding periods, according to Tukey's test (*P* <0.10); (*) no significant effect.

**Fig. 3:**
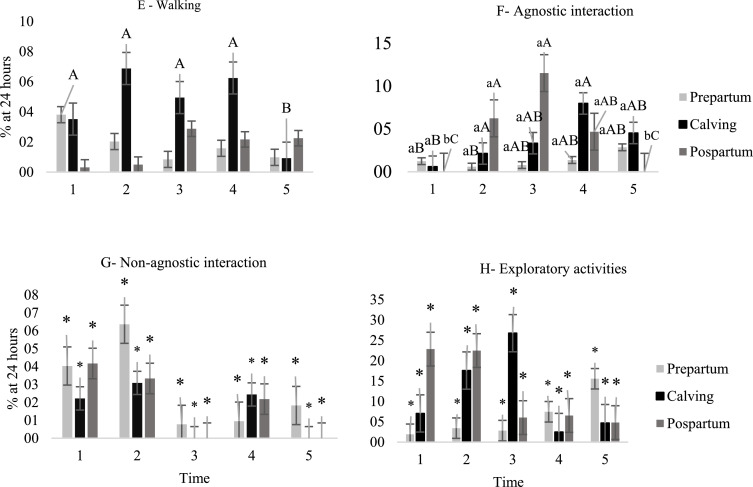
Behavior of goats evaluated one day before kidding, on the day of kidding and one day after kidding at different times of the day: (05:00–6:30 h); 2 (08:30–10:00 h); 3 (12:00–13:30 h); 4 (15:30–17:00 h) and 5 (19:00–20:30 h). Walking (E); agnostic interaction (F); non-agnostic interaction (G); animal in exploratory activities of the environment (H); time expressed as a percentage of 24 h. Lowercase letters differ for times and uppercase letters differ for delivery period according to Tukey's test (*P* <0.10); (*) no significant effect.

An interaction between the factors kidding and schedule was observed (*P* > 0.10) in the behavior of goats for the variables lying, feeding, and agnostic relationship (Fig. 3). Pregnant goats were lying for a longer time (*P* < 0.10; Fig. 2-C) 1 day before kidding, when they spent an average 30.5% of their time in lying at time schedules 1, 2, 3 and 4, and spent an average 46.2% of their time in lying at night (time schedule 5). Lying time significantly reduced on day of kidding, (*P* < 0.10).

Goats spent more time feeding at the time schedules 2 and 4 (*P* < 0.10), which is compatible with the feeding period (Fig. 2-A), regardless of the day of kidding. However, goats spent more time feeding on the days before and after kidding at time schedule 3. Additionally, for this same time schedule (3), and at time 4, feeding time was shorter on the day of kidding. At times 1 and 5, goats spent less time feeding, regardless of kidding (*P* < 0.10). No significant effect (*P* > 0.10) for rumination time (Fig. 2-B) was observed for the evaluated factors, when animals spent an average of 19.8% of the time ruminating.

In contrast, a higher proportion of agnostic interactions (Fig. 3F) were observed on the day of kidding and during the postpartum period, especially at times 2, 3, and 4. This time schedules are compatible with the feeding supply period (2 and 4) and times with higher temperature (Fig. 1).

**Fig. 4:**
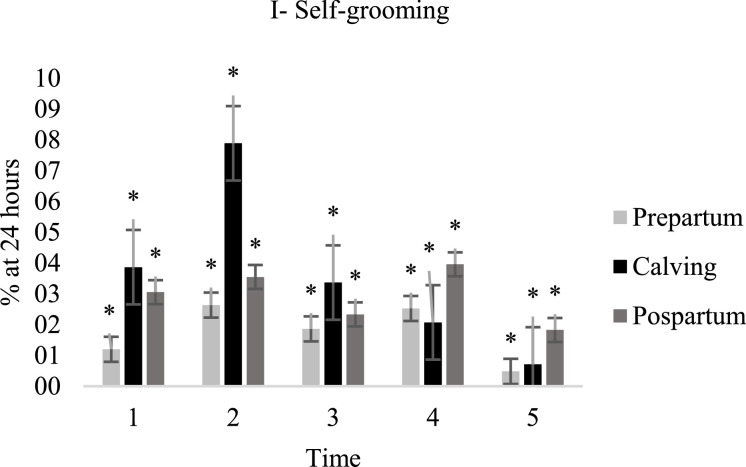
Behavior of goats evaluated one day before kidding (prepartum ), on the day of kidding (kidding ) and one day after kidding (postpartum ) at different times of the day: (05:00–6:30 h); 2 (08:30–10:00 h); 3 (12:00–13:30 h); 4 (15:30–17:00 h) and 5 (19:00–20:30 h). Animal in self-grooming (I), time expressed as a percentage of 24 h. Lowercase letters differ for times and uppercase letters differ for delivery periods according to Tukey's test (*P* < 0.10); (*) no significant effect.

Goats remained standing longer on the day before kidding (23.2%) when compared to the day of kidding (11.6%), and to the day after kidding (15.7%) (Fig. 2-D). At time schedule 2 goats spent more time standing, while they spent shorter times at schedules 3 and 5, while the other times did not differ from these. There was no effect (*P* > 0.10) of kidding, or interaction on moving behavior (Fig. 3-E). There was no effect (*P* > 0.10) of kidding, time schedule, or interaction on the non-agnostic, exploratory, and self-grooming relationships of the goats kept in collective bays (Fig. 3-G, H and Fig. 4-I).

**Fig. 5:**
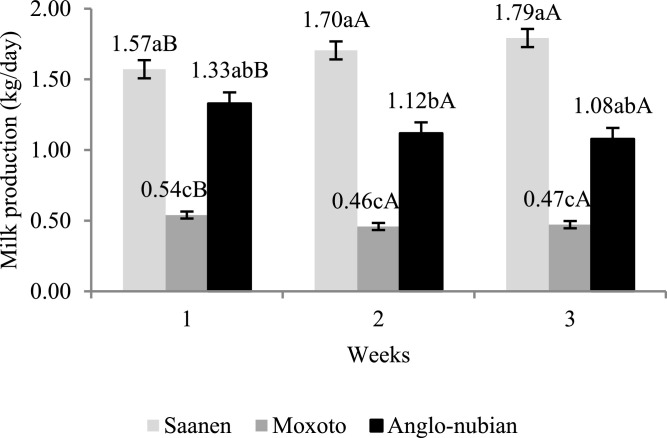
Milk production (kg/day) during the first 3 lactation weeks. Means followed by lowercase letters differ between breeds and averages, with capital letters differing for lactation weeks, according to Tukey's test (*P* < 0.10).

There was an effect of interaction (*P* < 0.10) between the genetic group and lactation week on milk production of the Saanen, Anglo-Nubian, and Moxotó goats (Table 2). The Saanen breed presented higher milk production during lactation in all lactation weeks studied, followed by Anglo-Nubian and Moxotó, which were intermediate and lower, respectively, inside each lactation week (Fig. 5).

**Table 2:** Milk production, milk fat and metabolite concentration in the blood of Saanen, Moxotó and Anglo-Nubian goats during the lactation period.

Item	Breeds			*SEM*	*P* value		
	Moxotó	Saanen	Anglo-nubian		Breed	Week of lactation	*B* × *W*
MP^+^	1.69 ^a^	0.49 ^c^	1.18 ^b^	0.28	<0.01	<0.01	0.08
MF*	3.92	6.60	4.92	0.05	<0.01	<0.01	<0.01
GL‡	58.80	58.0	57.3	0.54	0.60	<0.01	0.96
BHBA^†^	0.42	0.32	0.35	0.02	0.69	<0.01	0.11
TC ‡	88.3	81.0	78.2	1.10	0.28	<0.01	0.49
TRI‡	20.7	20.3	20.5	0.30	0.98	<0.01	0.69
VLDL‡	4.1	4.1	4.1	0.10	0.92	<0.01	0.58
HDL‡	28.2	27.6	27.9	0.40	0.99	<0.01	0.19
LDL‡	55.6	49.0	45.9	1.00	0.18	<0.01	0.23

^+^ Expressed in litter/day; * expressed in%; ^†^ expressed in mmol/l; ‡expressed in mg/dl. *SEM* standard error of media. MP milk production; MF milk fat; GL glucose; BHBA β-hydroxybutyrate; TC total cholesterol; TRI triglycerides; *VLDL* very low density lipoprotein; *HDL* high density lipoprotein; *LDL* low density lipoprotein. Means followed by lower case letters differ between breeds by the Tukey's test (*P* < 0.10).

There was no difference between breeds, and no interaction (*P* > 0.10) was noted between breeds and weeks of lactation on blood glucose. However, a difference (*P* < 0.10) was found between lactation weeks, when blood GL presented a peak concentration in Week 5, and a posterior decrease. Blood GL on the day of kidding (week 0) did not differ (*P* > 0.10) from lactation weeks 1, 2, 3, 4, 5, and 8.

There was no effect of genetic group and interaction (*P* > 0.10) on BHBA concentration (Table 2). The blood BHBA at week 1 was greater but did not differ (*P* > 0.10) from lactation weeks 3 and 4. There was no effect of genetic group and interaction (*P* > 0.10) on TC blood (Table 2). No difference (*P* > 0.10) was found on the day of kidding (week 0) when compared to lactation weeks 1, 2, 3, 4, 6, 7, and 8. At lactation week 5, the greatest total blood cholesterol was observed.

There were differences (*P* < 0.10) in the triglyceride, VLDL, HDL, and LDL serum concentrations during the lactation weeks (Table 2). On the other hand, there was no effect of genetic group or interaction on these variables (*P* > 0.10). The greatest value (*P* < 0.10) on blood triglyceride was observed at lactation week 4 (23.5 mg/dL), which did not differ from lactation weeks 1 and 6 (Table 2).

The greatest VLDL concentration was recorded at lactation week 8 (4.67 mg/dl), which did not differ (*P* > 0.10) from lactation weeks 0, 1, 3, 4, and 6. The HDL lipoprotein values were higher at lactation weeks 1, 4 and 7, and did not differ among lactation weeks 2, 3, 5, 6, and 8. The lowest HDL concentration was observed on the day of kidding (week 0), which did not differ (*P* > 0.10) from lactation weeks 2, 3, 5, 6, and 8. The greatest blood concentration of LDL lipoprotein was measured at week 5 (61.16 mg/dl) and did not differ from weeks 1, 6, and 7. The lowest concentration was 45.39 mg/dl (week 8), which did not differ (*P* > 0.10) from weeks 0, 2, 3, 4, and 6.

The blood calcium concentration of the Moxotó goats was greater (*P* < 0.10) than for the Saanen goats. However, there was no difference (*P* > 0.10) in serum calcium between the Anglo-Nubian goats and the other breeds (Table 3). The calcium concentrations from week 2 to week 8 were similar with an average value of 9.5 mg/dl. On the day of kidding (week 0) and at lactation week 1, the Ca concentration remained below 9 mg/dl. During the first week after kidding, the lowest (*P* < 0.10) calcium concentration was observed, which differed from the other weeks.

**Table 3:** Concentration of minerals, albumin, urea and total protein, aspartate aminotransferase and gamma-glutamyltransferase in the blood of Saanen, Moxotó and Anglo-Nubian goats during the lactation period.

Item	Breeds			*SEM*	*P* value		
	Moxotó	Saanen	Anglo-Nubian		Breeds	Weeks of lactation	*B* × *W*
Calcium^†^	9.4^a^	8.9^b^	9.1^ab^	0.09	0.04	<0.01	0.73
Phosphorus^†^ (mg/dl)	5.9	6.2	5.9	0.1	0.90	<0.01	0.65
Albumin^†^	5.4	3.8	4.7	0.3	0.39	<0.01	0.03
Urea^†^	35.4	38.1	35.6	0.6	0.23	<0.01	0.37
Total Protein^†^ (mg/dl)	5.2	5.3	4.9	0.1	0.10	<0.01	0.25
AST*	74.8	72.2	73.7	0.8	0.72	0.03	0.16
GGT*	33.7	35.3	33.7	0.3	0.45	0.42	0.19

†expressed in mg/dL; *Expressed in IU/L. *SEM* standard error of media. Means followed by lower case letters differ between breeds by the Tukey test (*P* < 0.05). AST aspartate-aminotransferase; GGT gamma-glutamyltransferase.

The blood phosphorus concentration differed (*P* < 0.10) between lactation weeks (Table 3). There was an increase during lactation weeks 6 and 7 (7.7 and 7.9 mg/dL, respectively), which differed from the other lactation weeks. The lowest concentration (*P* < 0.10) was observed at lactation week 3, which did not differ from weeks 0, 1, 2, 4, and 5.

No differences (*P* > 0.10) were observed in the blood concentrations of albumin, urea, and total protein for the evaluated goat breeds (Table 4). However, differences (*P* < 0.10) in lactation weeks were observed (Table 3). At week 1, the highest total protein value (5.77 mg/dL) was recorded, but it did not differ from lactation weeks 3, 4, 6, 7, and 8. There was a difference (*P* < 0.10) in the blood urea concentrations during the first 8 lactation weeks (Table 3). The urea concentration at lactation weeks 5, 6, and 8 (42.9, 41.0, and 42.6 mg/dl, respectively) were the highest and did not differ (*P* > 0.10) from weeks 3 and 7. The concentrations found on the day of kidding did not differ (*P* > 0.10) from weeks 1 and 2.

An effect on albumin concentration was noted for the week × breed interaction (*P* < 0.10). During week 6, the highest (*P* < 0.10) albumin values were found from Saanen than for the other breeds. Differences (*P* < 0.10) were observed in the albumin concentrations in the Saanen, Moxotó and Anglo-Nubian breeds at week 7 compared to the others.

No difference (*P* > 0.10) in the GGT enzyme concentration was observed among the evaluated genetic groups and an average of 73.6 IU/L was obtained. The lowest AST concentration in blood was observed on the day of kidding. The highest concentration was found during week 7. The concentration of the AST during lactation week 1 (74.4 IU/L) did not differ (*P* > 0.10) from the other weeks, except at week 0 (67.4 IU/L) (*P* < 0.10).

## 4 Discussion

Goats are social animals. Before kidding and at kidding, females tend to remain isolated, and they reduce their feed intake and movements (Edwards & Broom, 1982). Moreover, during gestation when kept in groups, individual spaces are reduced, and agnostic relationships increase, which may compromise animals’ behavior and welfare (Silanikove, 2000). This statement was confirmed by Vas and Andersen (2015). These author studied inter-individual distances, movement patterns and activity budgets in pregnant goats housed at different densities. They found that goats spent less time feeding and more time on resting and social behaviors on oncoming prepartum. They suggested that more space per goat is needed for goats closer to parturition than in the early gestation phase.

A greater proportion of agnostic interactions could be related to the need for isolation and a larger individual space, which could occur in goats at the time of kidding to guarantee their welfare. Krawczel and Lee (2019) observed increased social stress and aggression and reduced lying time in cows in higher stocking densities reported increased aggression behavior in goats at loose housing. Animals kept in limited areas and at high temperatures may have compromised its welfare (Conte et al., 2018). These findings could help to design more comfortable maternity pens for dairy goats.

Agnostic and discomfort relationships may be critical in pre-kidding, kidding, and postpartum females, because they contribute to increased risk of transition disturbs. In these phases, understanding the dynamics of females’ behavior can guarantee improved management practices and ensure the mitigation of stress in pregnant goats. According to our observations, the nature of the variation in goats’ feeding behavior was influenced by the feed supplying, with exception for time 3 (Fig. 2-A), in which feed intake lowered on the day of kidding. Reducing feeding at time 3 on day of kidding could be associated with the effect of hormonal discharge, which is released on the day of kidding and induces a reduction in feed intake.

Gandra et al. (2016) have emphasized that the transition period (late gestation and early lactation) is a critical physiological phase in the production system because of high nutrient requirements, caused by fetal growth, lactogenesis, and low dietary intake. Possibly the higher milk production observed in the Saanen goats led to a lower milk fat concentration in these animals. Queiroga et al. (2010) worked with crossbred Moxotó goats and observed negative relationships between milk production and milk fat content. Zamuner et al. (2020) related that postpartum fatty acids, BHB, and plasma urea nitrogen increased with increasing milk production.

The variations observed in the blood GL concentration of goats in this study probably not interfered in animals’ physiology, since normal values range between 50 and 74 mg/dL in goats (Kaneko, Harvey & Bruss, 2008). The preservation of normal glycaemia observed in the goats is probably associated with the physiological adaptation that occurs in ruminants during the NEB. In addition, when blood glucose concentration is adequate, adipose lipogenesis is favored over lipolysis, which results in a low concentration of non-esterified fatty acids (NEFA) in the blood. This effect could be observed in our results presented in Fig. 5 and Table 2, where the concentrations of energy metabolites are represented.

Thus, even with the observed changes in BHBA concentrations (Table 2) during the first 4 lactation weeks, goats could have maintained a positive energy balance during this period due to the BHBA values found (0.45 to 0.60 mmol/L), which were quantified within normal ranging. Radin et al. (2015) have stated that BHBA concentrations between 0.50 and 0.76 mmol/L would not imply any predisposition to metabolic disturbances in multiparous goats during the first 4 lactation weeks.

According to Ospina, Nydam, Stokol and Overton (2010), beta-hydroxybutyrate is the main ketone body produced by the liver and is a marker of the energetic balance. In general, an interaction exists between NEFA concentration and BHBA, where increased BHBA comes immediately after circulating NEFA increases (Cavestany et al., 2005). According to Edmondson and Pugh (2009), goats and sheep that present BHBA values between 0.8 and 1.6 mmol/L during the transition period are in NEB, with BHBA concentrations tending to increase during the postpartum period (Schlumbohm & Harmeyer, 2004), as observed in the present study. The BHBA values over 1.2 mmol/L are indicative of subclinical ketosis and may be related to a reduction in herd health and performance (Oetzel, 2004). Also, the BHBA resulted from this experiment could indicate an adaptation of the goats when concerning the postpartum energy balance. This may led to a lower risk of alteration in the energy metabolism of goats with similar milk yield.

Cholesterol values during the first 8 lactation weeks were below the values predicted by Tharwat and Al-Sobayil et al. (2013) for goats in the transition period (110 to 130 mg/dL). The low cholesterol concentration could be due to some oscillation in feed intake since the precursor for cholesterol synthesis is acetyl-coenzyme A, which becomes available through feed intake and increased insulin and leptin (Chilliard et al., 2001; Chilliard, Delavaud & Bonnet, 2005).

Radin et al. (2015) have reported blood concentrations of 0.09 to 0.19 mmol/dL of triglycerides in multiparous goats during the first 4 lactation weeks. These values were lower than those of the goats evaluated herein, which presented concentrations of 17.8 to 23.5 mmol/dL. These values found in this assay could be related to the efficiency of triglycerides usage by the mammary gland to synthesize milk fat. According to Mantovani et al. (2010) and Turk et al. (2013), at the early lactation in ruminants, the concentration of triglycerides in the blood is lower compared to pre-partum period, since the mammary gland use them for milk fat synthesis (Bernard, Leroux & Chilliard, 2008). This could help to confirm the hypothesis that low milk production goats probably did not present NEB.

The VLDL concentrations (3.58 to 4.67 mg/dL) suggest a very little mobilization of adipose tissue fatty acids, which led to a lower needing for lipids transformation by the liver, as well as for lipoprotein exportation. The present study observed less probability of animals presenting NEB, which reinforces the thesis of a minor need for the oxidation of fatty acids that derive from adipose tissue.

A 19.81% decrease in the calcium concentration took place during lactation week 1 (6.88 mg/dl) compared with the day of kidding (8.58 mg/dl). This drop can be considered physiologically normal. During the first lactation weeks, calcium concentrations tend to decrease due to increased milk production and colostrum synthesis. According to Goff (2000), the small number of receptors for 1,25 - dihydroxyvitamin D in the gut could lead to reducing calcium absorption during that period. This may explain the lowering calcium concentration observed during the first few postpartum weeks. Tharwat and Al-Sobayil (2013), evaluating goats during the transition period, observed a 20% drop in total calcium concentrations during the kidding week. The blood calcium during the first 8 lactation weeks agreed with the values found by Kaneko et al. (2008), who recommend around 8.5 to 11.7 mg/dL of calcium in goats. However, it went below that recommended by these authors during lactation week 1 (6.88 mg/dl). The phosphorus content during the first 8 lactation weeks fell within the normal range recommended by Kaneko, Harvey and Bruss (1997), between 4.2 and 9.1 mg/dL.

During the period close to kidding, the total circulating plasmatic proteins may drop below normal ranging due to immunoglobulin production to colostrum, and also due to fetus growing (Jainudee & Hafez, 1994). Excluding pathologic causes, the lesser circulation of total proteins in plasma or serum is associated with protein deficiency in the diet, which did not appear to have occurred in the present study, except on the day of kidding.

Urea diffuses through the alveolar epithelium of mammary glands into milk, and a high positive correlation has been reported between N-urea concentration in milk (NUL) and plasma N-urea (NUP) (Broderick & Clayton, 1997; Jonker, Kohn & Erdman, 1998; Roseler, Fergunson, Sniffen & Herrema, 1993). In this study, the highest blood urea concentrations were obtained after lactation peak, between weeks 5 and 8. At the end of gestation, blood urea content rose and these values lowered near, and soon after kidding, even with a balanced diet. Law et al. (2011) observed the same increasing urea effect when working with multiparous cows (40 animals) and primiparous (40) animals on high- and low-energy diets.

The AST is not considered a hepato-specific enzyme because it is present in liver cells (hepatocytes), and in larger amounts in the cardiac and skeletal muscles (Kaneko et al., 2008). In general, high AST concentrations are involved in hepatic injury, as these enzymes are not naturally occurring in plasma circulation. According to Thrall, Weiser, Allison and Campbell (2012), although AST is also present in muscle cells beyond the liver, its elevation during the transitional period may indicate greater liver activity. According to Tharwat and Al-Sobayil et al. (2013), increasing AST levels can be attributed to the accumulation of triglycerides in the liver due to the mobilization of body reserves to meet energy demands of the transition period. However, they did not perform a liver evaluation of triglycerides to confirm this relationship. In the present study goats probably presented a positive energy balance, which could lead to discarding the fact that triglycerides may be excessive in the liver, thus no conducting to AST increasing.

## 5 Conclusions

When dairy goats are kept in collective stalls (2 m^2^ per animal), they display exploratory and agnostic activity on the day of kidding, without this compromising welfare. However, we suggest that more space per goat is needed for goats closer to parturition than in the early gestation phase to avoid injuries and stress. Exploratory and agnostic activities could be inserted in the motoring routine of the periparturient herd, allowing to providing more space for this animals, improving welfare at kidding day.

The Saanen, Moxotó and Anglo-Nubian dairy goats adapted to tropical conditions of environment and feeding, with milk production up to 1.69 L/day do not enter NEB. This phenomenon can be associated with the reduced physiological adaptation requirement that occurred in this kind of herd during postpartum, when mobilization of adipose tissue fatty acids are reduced, leading to few alterations in energetic, protein and mineral metabolism. We recommend to constant motoring of milking goats after kidding avoiding excessive body weight gain, once low-medium producing dairy goats are not metabolically affected by NEB as the high producing dairy goats.

## Ethical statement

This study was conducted according to the guidelines of the National Council for the Control of Animal Experimentation. The Committee on the Ethics of Animal Experiments of the Federal University of Bahia (UFBA), Brazil, approved the protocol (Permit Number: 27/2014).

## Declaration of Competing Interest

The authors declare that are no conflicts of interest to the current manuscript.
